# Uncommon Glycosidases for the Enzymatic Preparation of Glycosides

**DOI:** 10.3390/biom5042160

**Published:** 2015-09-24

**Authors:** Antonio Trincone

**Affiliations:** Institute of Biomolecular Chemistry, National Research Council, Via Campi Flegrei, 34, Pozzuoli 80078, Naples, Italy; E-Mail: antonio.trincone@icb.cnr.it; Tel./Fax: +39-81-867-5095

**Keywords:** glycosyl hydrolases, glycosynthases, glycosides, enzymatic synthesis, biocatalysis

## Abstract

Most of the reports in literature dedicated to the use of glycosyl hydrolases for the preparation of glycosides are about gluco- (α- and β-form) and galacto-sidase (β-form), reflecting the high-availability of both anomers of glucosides and of β-galactosides and their wide-ranging applications. Hence, the idea of this review was to analyze the literature focusing on hardly-mentioned natural and engineered glycosyl hydrolases. Their performances in the synthetic mode and natural hydrolytic potential are examined. Both the choice of articles and their discussion are from a biomolecular and a biotechnological perspective of the biocatalytic process, shedding light on new applicative ideas and on the assortment of biomolecular diversity. The hope is to elicit new interest for the development of biocatalysis and to gather attention of biocatalyst practitioners for glycosynthesis.

## 1. Introduction

Carbohydrates can be isolated from natural sources and manipulated in different manners to produce useful molecules adapted for various applications. However, the true potential of these molecules is limited because of their complex structures, making their synthesis and modification very difficult. Special and elaborate chemistry has been developed for the synthesis of various glycosidic linkages characterizing this class of biomolecules. In the last fifty years, the advance in the chemical synthesis of glycosidic bond has represented one of the most successful aspects in organic synthetic chemistry for the intrinsic difficulties related to the yield and selectivity one has to face. In fact, even considering the formation of one single glycosidic linkage, multiple aspects must be considered: the reactivity of special leaving groups on monosaccharides acting as donors, the regioselectivity towards a single hydroxyl group on the acceptor molecule and the stereoselectivity in forming pure anomers [1]. The benefits of the use of enzymes in this field are based on the general advantages of biocatalysis: enzymes catalyze reactions with high rate accelerations over background; they are chemoselective and regio- and stereo-specific. This green approach is based on different classes of enzymes (aldolases, lipases, *etc.*) for the preparation of complex natural or unnatural saccharides, for molecular manipulation of hydroxyl groups, for functional group modification on specific positions, *etc.* However, glycoside hydrolases (endo- and exo-glycosidases) and glycosyltransferases are the most important classes of this portfolio of biocatalysts on which most of the enzymatic strategies are based. The widely-known former type is obviously useful also as a natural hydrolytic agent to avoid harsh conditions or the use of toxic elements characterizing chemical catalysis, starting from raw complex mixtures up to value-added simple materials [2].

Glycosidases are enzymes that normally break glycosidic bonds in glycoprocessing, but under certain controlled reaction conditions, the formation of glycosidic bonds is possible. This synthetic mode generally results in yields ranging from 20% to 40%. However, starting from the end of the last century, engineered glycoside hydrolases (glycosynthases) have represented an important biomaterial of great help for the improvement of the yields and efficiency of glycosylation reactions [3]. From the analysis of the last decade’s literature in the field [4], it resulted that research has been actively aimed at the uniqueness in terms of enzymatic features. Even less exploited, another important group is known as transglycosylases, which are highly related to their hydrolytic glycosyl hydrolases counterparts. This strict relation led to the conclusion that just subtle molecular adjustments, rather than major modifications, could act on the hydrolysis/transglycosylation ratio for many glycosyl hydrolases, as in a brilliant summing up that has been recently reported [5]. Among enzymes available to synthetic glycochemists, glycosyltransferases are less exploited for different reasons: (i) they require hardly-available nucleotide sugars as donors; and (ii) somehow, the heterologous production of these biocatalysts resulted in a difficult task.

In this review, rare examples of natural and engineered glycosyl hydrolases are reported. The description of their performances is made in terms of their natural hydrolytic action and their synthetic potential adopted in applicative fields. The discussion is developed from a biomolecular and a biotechnological perspectives, drawing attention to possible new interests in modified enzymes from specific wild-type examples, thus increasing the attention of biocatalyst practitioners.

## 2. Glycosyl Hydrolases: Mechanisms, Use in Synthesis and Rare Examples

Enzymes hydrolyzing glycosidic linkages (glycosyl hydrolases) are considered one of most capable biocatalysts in that chemical spontaneous hydrolysis of unactivated *O*-glycosides proceeds much more slowly than the hydrolysis of bonds joining other biological polymers [6]. The stereochemical outcome of the enzymatic reaction can furnish products with inversion or retention of the anomeric configuration with respect to starting substrates. Enzymatic reaction mechanisms have been long known [7]. Retaining glycosidases maintains the anomeric configuration of the substrate in the products, via a double displacement mechanism. Inverting glycosidases induce inversion, in a one-step reaction. However, other mechanisms making use of a different chemistry are known today [8]. The elimination-hydration mechanism, involving the presence of NAD^+^ and mercaptoethanol, is of particular importance for the formation of a glycal intermediate [9]. Although it seems not to be reported in literature as a possible transglycosylation mechanism, in the case of 6-phospho-β-glucosidase from *Thermotoga maritima*, it was proven that transglycosylation activity was responsible for the formation of methyl 6-phospo-β-glucoside in the presence of methanol as an acceptor [10].

For the use of glycosyl hydrolases in the synthesis, two experimental protocols are possible: the reverse hydrolysis procedure and the kinetic approach. The first, using a free monosaccharide and an acceptor, is reported as an efficient and cost-effective methodology only for some enzymes. It is a thermodynamic approach, whose equilibrium is shifted towards the products by using different methods: high acceptor concentration; the use of cosolvent and a temperature increase. Generally, yields are between 40% and 60% in optimal cases. The alternative kinetic approach, based on the use of a glycosyl donor forming an intermediate glycosyl enzyme, is also possible. The intermediate in the presence of an acceptor is resolved in the products freeing the enzyme. If the acceptor is not water, the products are still substrates for the enzyme; hence, this transglycosylation reaction must be carefully monitored to achieve a good yield [11].

The stereochemical outcome of the synthesis reaction as concerns regioselectivity depends on the nature of the enzyme and on its relative activity for the donor and the products formed. Hence, the choice of donor substrates is an important issue in transglycosylation reactions. Donor engineering has been an interesting topic in recent years in the field [12], moving beyond *O*-glycosides as the sole substrates. Indeed, many others have been added to the list for the preparation of modified carbohydrate products. How the use of various donors influences the regioselectivity or the yield of the enzymatic reactions has been a topic of choice. Usually, as activated and more efficient substrates (*i.e.*, possessing suitable kinetic parameters), aryl glycosides are considered: phenyl, nitrophenyl or the most recently found nitropyridyl substrates are characterized by great efficiency in the transfer [13].

The different types of glycosidases are classified using different systems. One is the specificity for the glycon structure of their substrates, although it can be very often relaxed [14]. Enzymes from different sources can hydrolyze different glycopyranosides, and this is the case for bovine liver β-galactosidase or *Pyrococcus furiosus*, *Sulfolobus solfataricus* and *Agrobacterium* sp. β-glycosidases. These preferences are expressed by their relative activity against substrates having various aglycons [12].

The most abundant carbohydrate is d-glucose found as a free “sugar” in honey, plant nectars and fruit juices, but occurring usually in a glycosidically-bound form and metabolized as phosphate ester. In the approximate order of natural abundance, isomers of glucose are found as follows: the keto-sugar d-fructose and the stereoisomers of glucose; d-galactose and d-mannose. In a very superimposable manner, scientific knowledge of related glycosidically-active enzymes has been historically acquired. In Nature, along with the above-mentioned monosaccharides, the C6-oxidized derivatives, d-glucuronic, d-galacturonic and d-mannuronic acids, are found. Five-carbon sugars, l-arabinose and d-xylose, are abundant in plant tissues (pectins and hemicelluloses), while d-ribose and 2-deoxy-d-ribose are constituents of RNA and DNA, respectively. Both 6-deoxy sugars l-rhamnose, stereochemically-related to l-mannose, and l-fucose related to l-galactose, are components of pectins, xyloglucans, mucilages and glycoconjugates. Critically important for its biological activity is a mannose derivative, *N*-acetylneuraminic acid, one member of the extensive family of sialic acids. Rare, but important sugar derivatives are constituted by a seven-carbon chain. Antibiotics and steroidal glycosides contain sugar units modified by specific changes of functional groups. Well over a hundred modified sugars, including many methyl ethers, and ketoacids are known to be present in constituent units in bacterial polysaccharides, and also in these cases, the knowledge about enzymes acting on these molecules parallels their scarce natural abundance, as observed above.

l-arabinose is mobilized by arabinofuranosidases, which is the first class of enzymes discussed here. As a quick indication of their uncommonness, the search in titles and abstracts for “glucosidase*” and “galactosidase*” from 1994 to 2015 in the Science Direct database sorted out approximately ten-thousand different articles, while “arabinofuranosidase*” and “arabinosidase*” both collected no more than three hundred products. Xylanases and xylosidases acting on d-xylose are also included in this review with emphasis on the lesser known α-xylosidases. These biocatalysts are involved in the degradation of plant biomass. l-sugar rhamnose is found in many natural products, and rhamnosidases are included in this review. In fact, there are very few articles reporting the transfer action of the rhamnosyl group for these enzymes. Another important sugar, such as l-fucose, is present in important molecules, and the enzymes related in bioprocesses involving it are grouped here under the dedicated paragraph. For galactose, the analysis is limited to α-galactosidases and their importance in biomedicine. Among the paragraphs including very few examples, glucuronidases, hyaluronidases and sialidases are mentioned here for the importance of the molecules on which they act and their long-known involvement in a variety of biological processes. Inulinases are also added for their importance in the food domain of the transformed molecules.

## 3. Arabinofuranosidase

d-xylose and l-arabinose are the first and second most abundant pentoses found in plant polysaccharides. l-arabinose is present in arabinoxylan, arabinogalactan and arabinan. The complete degradation of these polymers requires the concerted action of many different hemicellulose-hydrolyzing enzymes. Arabinanases catalyze the hydrolysis of the backbone of the arabinan in an endo-manner. Exo-acting and retaining α-l-arabinofuranosidases release terminal non-reducing α-l-Ara*f* residues from different α-l-arabinosyl-containing polysaccharides. Enzymes of this kind are found in the families GH2, 3, 43, 51, 54 and 62 of the CAZy database [15] and are named according to their hydrolytic specificities. They are naturally involved in the degradation of plant biomass and of other l-arabinose-containing compounds.

**Figure 1:**
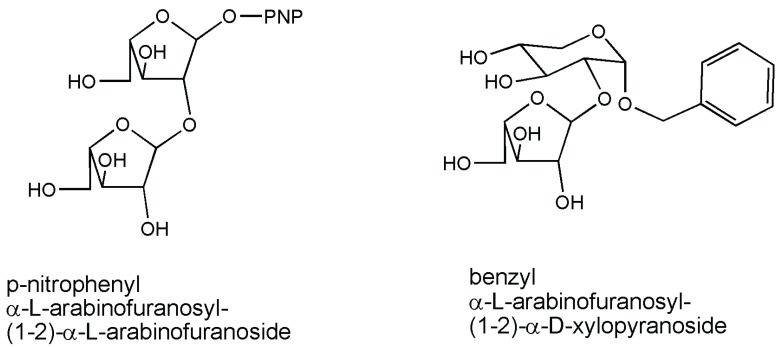
Arabinofuranosides synthesized by thermophilic α-l-arabinofuranosidase from *Thermobacillus xylanilyticus* [17].

A general investigation of transglycosylation capability possessed by an interesting thermophilic α-l-arabinofuranosidase from *Thermobacillus xylanilyticus* was already reported in 2002 [16]. The use of different alkyl alcohol acceptors was tested, discovering that primary and secondary alcohols acted efficiently. Yields decreased with increasing alkyl chain length. The same enzymatic activity was exploited for the synthesis of oligosaccharide motifs in that they could be used as reference substrates for the study of hemicellulases, namely pNP-α-l-arabinofuranosyl-(1-2)-α-l-arabinofuranoside (Figure 1). 1,3 and 1,5 isomers of this disaccharide were also formed in very small proportions. When using benzyl α-d-xylopyranoside (Ph-CH_2_-α-Xyl*p*) as the acceptor, one of the disaccharides produced was benzyl α-l-arabinofuranosyl-(1-2)-α-d-xylopyranoside (Figure 1), but other arabinobiosides resulting from the autocondensation of pNP-Ara*f* were also produced [17]. The same α-l-arabinofuranosidase from *Thermobacillus xylanilyticus* was also reported as a catalyst in the synthesis of β-d-galactofuranosides owing to the structural similarity between d-Gal*f* and l-Ara*f*. Galactofuranose-based glycoconjugates are interesting target molecules for drug design, being galactofuranose totally absent in mammals. However, the specificity ratio of the enzyme for arabinofuranoside and galactofuranoside linkages was measured to be 465/0.64 IU/mg, respectively. Autocondensation of p-nitrophenyl-β-d-galactofuranoside, forming the dimer p-nitrophenyl β-d-galactofuranosyl-(1-2)-β-d-galactofuranoside, and transgalactofuranosylation of benzyl α-d-xylopyranoside, forming benzyl β-d-galactofuranosyl-(1-2)-α-d-xylopyranoside, were both reported [18]. In the second case, a yield of approximately 75% was indicated. Both reactions were scaled up to a 12-mL final volume, using 500 IU of enzyme at 5 mM pNPGal*f* alone or in combination with an equimolar amount of the benzyl xylopyranoside acceptor. Substrate analogs modified at C-5 (5-deoxy or 5-F) or C-2 (2-deoxy) were also subsequently considered for reactions [19], while enzyme versatility was also proven on other substrates [20]. Finally, to detect transglycosylating mutants, random mutagenesis using a high-throughput digital imaging screening methodology was performed on the wild-type catalyst to enhance its ability to perform transarabinofuranosylation using natural xylo-oligosaccharides as acceptors. The reactions catalyzed by the most promising mutants revealed a significant improvement of the yields of transglycosylation products from 9% (wt) to 18% with the xylobiose acceptor [21], while up to a 96% yield was obtained in the presence of various aliphatic alcohols [22]. It is of certain interest that with this α-l-arabinofuranosidase from *Thermobacillus xylanilyticus*, some fundamental details concerning pH modulation by amino acid residues in the protein were revealed [23]. General outlined conclusions of the authors toward the applicability of the results to other glycoside hydrolases suggested the importance of the effects of mutations at position 344 on the transglycosylation/hydrolysis partition and could provide clues useful for further engineering toward efficient transfuranosidases/transglycosidases.

**Figure 2:**
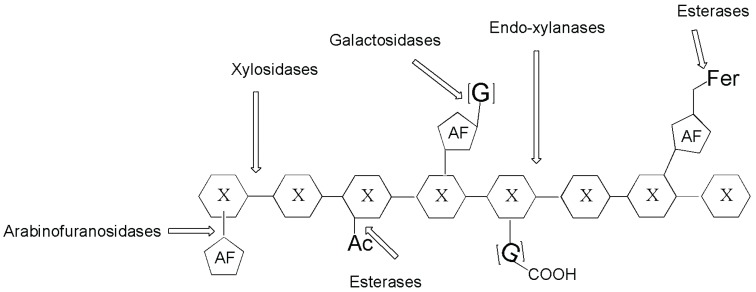
Schematic representation of enzymes attacking xylan chains. Arabinofuranosidase as one of the enzyme for the complete hydrolysis of xylan. X in the hexagon is the symbol used for xylose; AF in the pentagon is for arabinofuranose; G is galactose; Ac is the acetyl group; G-COOH is glucuronic acid.

As indicated above, arabinofuranosidases are among the accessory enzymes for the conversion of pretreated lignocellulose and can be used to transform the biomass into fermentable sugars (Figure 2). In studies oriented toward this practical domain, a fungal arabinofuranosidase from *Pleurotus ostreatus* recombinantly expressed in *Pichia pastoris* was tested to enhance the enzymatic saccharification of lignocellulosic biomasses [24].

**Figure 3:**
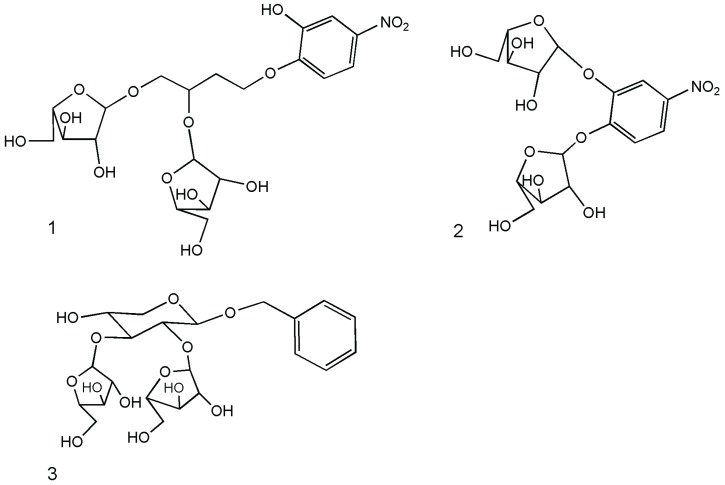
Artificial substrates enabling the study of selectivity of arabinofuranosidases [25].

Fine substrate recognition was studied for the GH43 enzyme from *B. adolescentis* using two chromogenic artificial substrates enabling one to demonstrate how the selectivity of this enzyme is a property that often remains unstudied due to the lack of appropriate substrates and readily-accessible methods. In an elegant experiment, enzyme-mediated hydrolysis of 1, 2 and 3 (Figure 3) was monitored by ^1^H NMR, performing reactions in standard 5-mm NMR tubes [25], taking advantage of the clear differences in the chemical shifts of the anomeric signals of different arabinose moieties at different positions.

It is of interest to note that in a recent case, the extracellular α-l-arabinofuranosidase purified to homogeneity from *Talaromyces thermophilus* was found to be active on α-linked arabinoside in the furanoside configuration retaining also 52% of its activity in the presence of p-nitrophenyl-β-d-xylopyranoside as the substrate [26].

From another thermophilic species, a new enzyme with β-xylosidase activity was discovered and cloned in *E. coli*. Along with the appreciated possibility of using it without inhibition in the presence of up to 1 M xylose in synthetic mode, the enzyme exhibited also interesting activity on p-nitrophenyl-α-l-arabinofuranoside [27].

In contrast to α-l-arabinofuranosidases, β-l-arabinofuranosidases have been discovered only recently [28]. The sugar β-l-arabinofuranose is present in terminal positions in plant biopolymers, in hormones, *etc.* Four differently-linked units are found attached to hydroxyproline (Ara4Hyp) in glycoproteins, and one of the enzymes related to the catabolic system is also able to catalyze the transglycosylation to 1-alkanols [29].

## 4. Rhamnosidase

Rhamnose is a naturally-occurring l-sugar. It is found as the glycon part in many natural products. It is also present in polysaccharides of (bio)medical importance. Although actively studied, there are very few articles reporting the enzymatic transfer of the rhamnosyl group. The first isolation of a rhamnosidase (naringinase) was reported in the literature in 1938, with fungi being the main source of these biocatalysts. However, these enzymes have been isolated also from animal tissues, such as the liver of both the marine gastropod *Turbo cornutus* [30] and pig [31], as well as from plants, such as *Rhamnus daurica* [32] and *Fagopyrum esculentum* [33], in addition to bacteria. Rhamnosidases cleave the rhamnosyl moiety linked to various groups and are very important biocatalysts from a biotechnological point of view for applications in debittering fruit juices, in the process of the enhancement of wine aromas and in the derhamnosylation of many natural products containing terminal α-l-rhamnose to access aglycones of pharmaceutical interest, as well as rhamnose itself. As recently reported, bioconversion of flavonoids is possible [34] by using these enzymes.

A fungal diglycosidase was recently isolated from *Acremonium* sp. It has an α-rhamnosyl-β-glucosidase activity with transglycosylation potential of the entire disaccharide (rutinose) moiety from natural products to different acceptors. The biocatalyzed synthesis of 4-methylumbelliferyl-rutinoside has been possible. The use of this fluorogenic substrate with the rutinoside moiety enables the study of this type of enzyme. Although with a modest yield (16% with respect to the sugar acceptor) [35], this remarkable, simple, one-step enzymatic synthesis was performed using hesperidin as the rutinose donor.

The finding that rhamnose does not penetrate into the skin, while rhamnosides (e.g., pentyl rhamnoside or higher, e.g., undecyl rhamnoside) reached active concentration in stratum corneum and epidermis with respect to free sugar, which increased the attention for compatible preparation methods of rhamnosides for applications in cosmetics or the food industry. In the research for this compatibility, a recent study reporting a chemo-enzymatic synthesis of α-l-rhamnosides by recombinant α-l-rhamnosidase originating from *Aspergillus terreus* is of extreme interest [36]. The reverse hydrolysis approach used for rhamnosylation was studied and optimized for the temperature, concentrations of the substrate, the nature of the solvent and the concentration. The syntheses of cyclohexyl, anisyl and 2-phenylethyl α-l-rhamnopyranosides were described, and rhamnosylation of phenolic hydroxyls of hydroquinone, resorcinol, catechol and phenol itself was also possible. Commonly-used solvents, such as DMSO, polyethylene glycol, acetone, 40% dimethylformamide, acetonitrile and tert-butyl alcohol, were necessary for rhamnosylation. In particular, the easily removable acetone, exhibiting excellent solubilizing properties, was highlighted as an optimal cosolvent. Final product concentrations from 2 to 10 g/L were obtained using C5 and C6 alcohols. Various monoterpenoids were also rhamnosylated, as well as anisyl alcohol, cinnamyl alcohol and 2-phenylethanol, making the system of interest in flavor and fragrance biocatalysis. Glycosylation was performed in the presence of 1.5 M rhamnose and 20 U/mL α-l-rhamnosidase.

**Figure 4:**
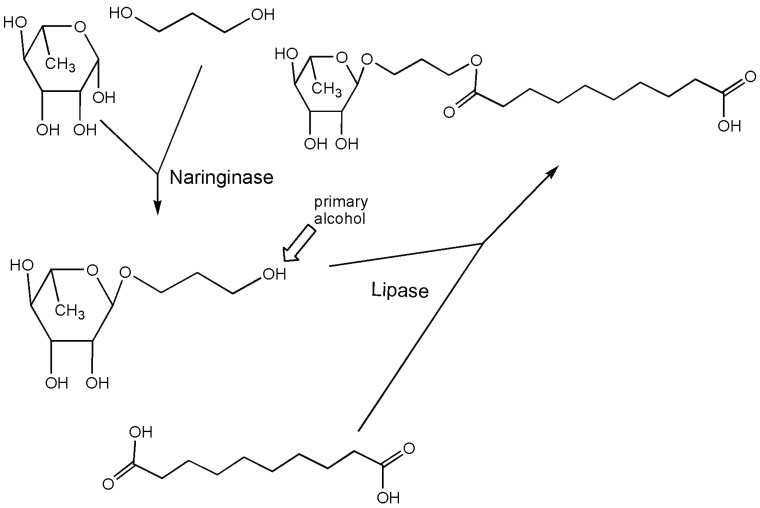
Biocatalytic two-step production of model rhamnolipid-like structures [37].

Other important biomolecules possessing rhamnose are the rhamnolipids constituted by one or two rhamnoses and a hydrophobic chain with 1 to 3 β-hydroxy fatty acids. Rhamnose is glycosidically-linked only to a secondary hydroxyl group of one of the fatty acids. In a reported biocatalytic step to produce this general kind of molecular model (rhamnose possessing a long hydrophobic chain), a naringinase was used to rhamnosylate 1,3 propanediol. This product, possessing a rhamnosyl moiety on the primary hydroxyl group, can easily be acylated by other enzymes [37] to produce novel rhamnolipids (Figure 4). The use of naringinase was already known for rhamnosylation using both rhamnose as a free sugar or naringin as a donor [38]. However, for the last reaction, the authors recognized that their results suggested a glycosylation of the diol only after the hydrolysis of naringin, acting by a reverse hydrolysis using the generated rhamnose. Yields strictly depended on the chain length of the acceptor with a dramatic drop of an order of magnitude from one (methyl rhamnoside, approximately 70%) to three carbon atoms.

**Figure 5:**
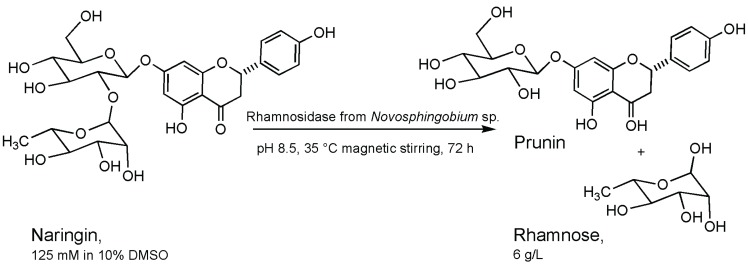
Naringin hydrolysis producing prunin and rhamnose [34].

Indeed, in a recent paper some, light was shed on the type of enzymatic mechanism of a new enzyme from the marine isolate *Novosphingobium* sp. studying the hydrolysis products of the aryl rhamnoside substrate by ^1^H-NMR spectra over time by the integration of the anomeric proton signals of the reagent and products. The kinetic behavior suggested an inverting mechanism of hydrolysis in which β-rhamnose was directly formed from the α-rhamnose via a single displacement mechanism. The activity reported was able to hydrolyze several flavonoids of interest for the pharmaceutical and food industries, and *Novosphingobium* sp. still represent a yet unexplored reservoir of interesting rhamnosidases, which might show novel interesting properties [34]. Data concerning hydrolytic features suggested that this α-RHA activity was able to hydrolyze both α-1-2 and α-1-6 interglycosidic linkages. At the best experimental conditions found, exploiting its alkaline pH optimum, starting from a 125 mM naringin solution, prunin was produced with a yield of 32.1%. Furthermore, free l-rhamnose was produced as a secondary product at a concentration of 6 g/L (Figure 5).

The yield of products obtained in the above experiment was comparable to that reported for a recombinant α-RHA from *Clostridium stercorarium* used for the hydrolysis of naringin from citrus peel waste [39] and for other similar processes [40].

Likewise, a hydrolytic process was reported for a fungal α-rhamnosidase. Rutin was subjected to enzymatic derhamnosylation to isoquercetin. The extracellular α-l-rhamnosidase of *Aspergillus terreus* was also expressed in the yeast *Pichia pastoris* as a fully-functional protein. Both the native and the recombinant α-l-rhamnosidases catalyzed the conversion. The product is an important flavonoid in nutraceutics possessing better absorption and bioavailability. This procedure has high volumetric productivity (up to 300 g/L). The complete absence of glucosidase activity secured the null complete hydrolysis to quercetin [41].

In another very interesting hydrolytic approach, rhamnosidase activity (from *Alternaria* sp. L1) was cloned and anchored on the cell surface of *S. cerevisiae*. With such a biocatalyst, approximately 80% of naringin in the juices can be hydrolyzed, suggesting it as a useful tool for debittering grapefruit juices [42].

## 5. Fucosidase

Another important l-sugar is fucose with α-l-fucosidase as the related enzyme and with examples both in hydrolysis and in synthesis. Very recently, the role of α-l-fucosidase *in vivo* has been seen under new light besides its digestive function. In an analyzed case, it has been considered important in host/parasite interactions [43]. As previously known, the deficiency of this enzyme results in fucosidosis, a lysosomal storage disorder [44]. Few examples of enzymatic synthesis of fucosyl glycosides were reported (see also Table 1).

**Table 1:** Enzymatic transfucosylation examples.

Enzyme	Products	References
α-l-fucosidase (porcine liver)	α-l-(1-2)- and α-l-(1-6)- fucosyl derivatives of methyl β-d-Galp; 1,1-trehalose-type disaccharide with Glc-*N*-Ac.	[45]
α-l-fucosidase (porcine liver)	O2, O3, O6 fucosylations of galactose of β-d-Gal-(1-4)-d-GlcNAc.	[46]
α-l-fucosidase *Aspergillus niger*	3-*O*-fucosyl disaccharides with 2-acetamido-2-deoxy-d-glucose and glucose.	[47]
Fucosidase *Corynebacterium* sp. and *Ampullaria*	methyl 2-*O*-α-l-fucopyranosyl-β-d-galactopyranoside.	[47]
Fucosidase *Ampullaria*	methyl 6-*O*-α-l-fucopyranosyl-β-d-galactopyranoside	[47]
α-l-fucosidase *Alcaligenes* sp. strain KSF-9687	*O*-3-fucosylation of galactose of β-d-Gal-(1-4)-d-GlcNAc; *O-*3-fucosylation of galactose of lactose; *O-*3 fucosylation of β-d-Gal-(1-4)-d-GlcNAc-OMe, β-d-Gal-(1-4)-d-Glc-OMe, β-d-Gal-(1-3)-d-Glc-OMe.	[48]
α-l-fucosidase *Alcaligenes* sp. strain	*O-*3-fucosylation of galactose in PNP-β-lactose and PNP-β-lactosamine.	[49]
α-l-fucosidase bovine kidney and testes	*O-*4-fucosylation of (6-*O*-Bn)-Glc-NH_2_-β-SEt.	[50,51]
α-l-fucosidase *Penicillium multicolor*	3-*O*-fucosyl disaccharide with 2-acetamido-2-deoxy-d-glucose and methyl or allyl derivatives.	[52]
α-l-fucosidase *Sulfolobus solfataricus*	3-*O* and 2-*O* fucosylation of PNP-β-d-glucoside.	[53]
α-l-fucosidase *Pecten maximus*, canine α-l-fucosidase	highly branched fuco-oligosaccharides as large as tetrasaccharides.	[54]
α-l-transfucosidase variants α-l-fucosidase *Thermotoga maritima*	*O*-3-fucosylation in self-condensation of PNP-fucoside *O-*2-fucosylation to PNP-Gal	[55]

In 1990, Svensonn and Thieme described the synthesis of α-l-(1-2)- and α-l-(1-6)-linked fucosyl derivatives. They used an α-fucosidase isolated from porcine liver [45].

**Figure 6:**
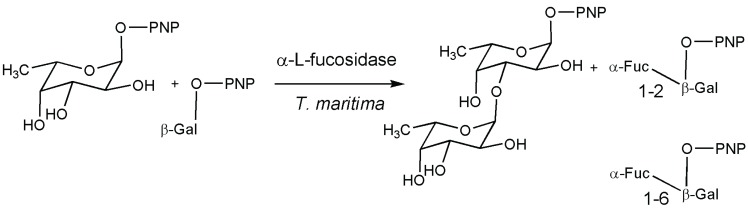
1,3-difucosyl product formed by α-l-fucosidase of *Thermotoga maritima* and fucosylated galactosides in 2 and 6 positions [55].

Produced by a mutation of the wild-type α-l-fucosidase from *Thermotoga maritima*, a transfucosidase variant of this enzyme deserves to be mentioned. A specific transglycosylation product, pNP-Gal-α-(1-2)-Fuc, reached more than 60% in yield compared to 7% with the wild-type enzyme at 10 mM concentrations of the donor and acceptor [55] (Figure 6). An interesting discussion based on molecular modeling calculations, in order to understand how a sugar acceptor could affect the transfucosidase regioselectivity, is reported. The unique conformation of the 1,3-difucosyl product is compatible with the active site topology, while for fucosylation of the galactose moiety, only one conformation of a (1-2)-substituted product is preferred inside the catalytic site.

Another important example to be cited is the α-l-fucosidase expressed by a single insertional mutation in the region of overlap between the ORFs SSO11867 and SSO3060 of the hyperthermophilic archaeon *Sulfolobus solfataricus* [53]. This enzyme, belonging to glycoside hydrolase family 29 (GH29), showed micromolar specificity for p-nitrophenyl-α-l-fucoside (pNP-Fuc) and promoted the transfucosylation reaction. It catalyzed the formation of the α-(1-2) and α-(1-3) bonds between fucose and glucose of pNp-Glc. The α-anomeric configuration of the interglycosidic linkages in the products unequivocally showed that GH29 α-fucosidases follow a retaining reaction mechanism. With no efforts made for the optimization of the reaction conditions (just keeping as low as possible the acceptor/donor equivalent ratio useful for synthetic application with rare acceptors), a 14% yield of fucosylated products was obtained. Moreover, the hydrolytic activity observed using α-l-Fuc-(1–3)-α-l-Fuc-*O*-pNP, liberating the external fucose in the sequence, revealed that Ss-α-fuc is an exo-glycosyl hydrolase that attacks the substrates from their non-reducing end.

The enzymatic formation of the β-d-fucosides is also hardly described in the scientific literature, and it deserves to be mentioned here. Acceptor specificity for monosaccharides and transfer efficiency have both been investigated for a glycosidase isolated from the China white jade snail [14]. An extraordinarily broad substrate specificity for both hydrolysis and transglycosylation was exhibited by this biocatalyst, and from the results obtained, the authors indicated a very high transfucosylation efficiency using p-nitrophenyl β-d-fucopyranoside at 10 mM and various acceptors (at 20 to 100 mM), such as glucose (88% yield) and xylose (93% yield); the interglycosidic linkage formed with glucose was β-1,6; thus, they proposed this biocatalyst as a useful candidate for Fuc-β-Glc and Fuc-β-Xyl disaccharide synthesis. The Fuc-β-Xyl building block formed is present in natural asterosaponins of marine origin, although in the natural compound, the interglycosidic linkage is different (Fuc-β-(1-2)-Xyl) [56].

## 6. Xylanase and Xylosidase

Hemicelluloses, mostly composed of xylans and xyloglucans, are assembled *in vivo* with cellulose in a network of cross-linked fibers. While xylans are heteropolymers consisting of a backbone of β-(1,4)-linked d-xyloses with various branching saccharidic groups (e.g., glucuronic acid, arabinose, *etc.*; see also Figure 2), xyloglucans, widely distributed in plants, have different structures composed of a β-(1,4)-glucan backbone, with α-(1,6)-d-xylose linked to about 75% of the glucosyl residues. The complete degradation and recycling of these polysaccharides involve different enzymes. Prevalent xylanases and β-xylosidases act on xylan (see briefly below), while lesser known α-xylosidases act on xyloglucan or on isoprimeverose (α-d-xylopyranosyl-(1,6)-d-glucose), a disaccharide that can be considered the building block of xyloglucan chains.

A thermophilic α-xylosidase from the archaeon *Sulfolobus solfataricus* was cloned and expressed in *E. coli* by Moracci *et al.* [57]. The enzyme showed high specificity for the hydrolysis of isoprimeverose and other oligosaccharides of xyloglucan and possessed transxylosidic activity, which was exploited in the synthesis of isoprimeverose-based compounds. As a donor, pNP-α-d-xylopyranoside was used in the presence of two-fold molar excess of pNP-β-Glu as the acceptor. The pNP derivative of isoprimeverose, an important chromophoric substrate for this class of enzymes, was regiospecifically synthesized with an 18% yield. Additionally, for the synthesis of free isoprimeverose, glucose as an acceptor was used, and yields ranging from 10% to 16% were obtained. With the aim of constructing trisaccharidic chromophoric derivatives of xyloglucan, pNP-β-cellobioside was also used as an acceptor. In this case, α-xylosyl fluoride was adopted as the glycosyl donor. Only three of the seven trisaccharidic possible compounds were formed in a 15% yield. In all of them, xylose units were placed on external glucose of the pNP-β-cellobioside acceptor, demonstrating that the exo-acting characteristic of this enzyme found in the hydrolytic reaction is also operative in synthetic mode [58].

**Figure 7:**
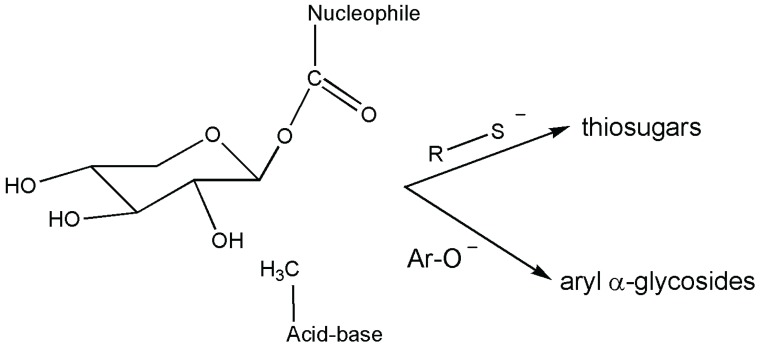
Schematic indication of the thioglycoligase intermediate in the synthesis of thiosugars and aryl glycosides.

An *E. coli* α-xylosidase mutant (YicI-D482A) efficiently catalyzed the synthesis of *O*-aryl α-xylosides in quantitative yields (up to 99%) using 4-methylumbelliferone or nitrophenols. An α-glucosidase mutant MalAD416A of the α-glucosidase of *S. solfataricus* also formed aryl glucosides of 3,4-DNP, 3-NP, 4-NP and MU used as sugar acceptors [59], as reported in the same paper. Thioglycoligases represents a further step in this field with respect to previously devised glycosynthases with nucleophile amino acid substitution. By modification of their general acid/base catalytic residue with an inactive amino acid, retaining α-glycosidase mutants can catalyze the formation of S-glycosidic linkages using a sugar donor with an excellent leaving group and a suitable sugar acceptor with a thiol group, thus the name thioglycoligases. Using thioglycoligases, the synthesis of *O*-aryl α-glycosides is also possible when aryl compounds possessing a hydroxyl group with a pKa value lower than normal sugars are adopted as the sugar acceptor (Figure 7), resulting in the formation of *O*- instead of the S-glycosidic linkages. Both α-thioglycoligases derived from the two retaining α-glycosidases mentioned above were used for the synthesis of *O*-aryl compounds of high interest as chromophoric substrates, such as methylumbelliferyl or 3-nitrophenols.

Scarce exploitation of products related to the field of pentose-specific conversion/utilization technologies [60] and the related slow development of inherent enzymatic processes caused neglect of the importance of heteroxylan from lignocellulosic material in biorefineries. The specific action of xylanases on xylan can lead to oligosaccharides, and it seems important to mention here some xylanase and β-xylosidase exploitable in this context. Using xylan, obtained after dilute alkaline extraction from raw materials, the hydrolysis was studied using a variant of the alkali-tolerant *Bacillus halodurans* S7 endoxylanase A (K80R), for the production of xylooligosaccharides. The enzyme was optimally active at 60 °C at pH 9, and the process featured a low xylose production (2.4%), which is desirable for XOS-enrichment during extended incubation. Within 7 h, a 36% conversion was achieved with xylan predominantly converted to xylobiose [61].

As for this specific hydrolytic action of xylanases, of interest are other recently-reported activities: a multifunctional enzyme from *Bifidobacterium longum* subsp. *infantis* ATCC 15697 [62] and the biocatalysts from *Thermotoga neapolitana*, *Geobacillus thermantarcticus* and *Thermoanaerobacterium thermostercoris*, recently applied in processing hemicelluloses from rhizome of *Arundo donax* [63]. Arundo’s rhizome represents a useful raw material for the production of valuable industrial products, and the study aimed to increase the economic efficiency of *A. donax* cultivation. Xylobiose and further interesting tetra- and penta-saccharide were obtained in different conditions. A convenient amount of raw material was processed per mg of crude thermophilic proteins.

The synthetic aspect of xylosidases in the synthesis of xylooligosaccharides has also been reported. Two β-xylosidases from *Aspergillus nidulans* FGSC A4 with different catalytic efficiency were produced in *Pichia pastoris* and secreted into the culture supernatants in high yields (16 and 118 mg/L). Transxylosylation was studied using nine monosaccharides, seven disaccharides and two sugar alcohols as acceptors, obtaining 18 different β-xylosyl-oligosaccharides synthesized in a 2% to 66% yield depending on the enzyme and substrate. Specificity for monosaccharide acceptors resulted for d-mannose, d-lyxose, d-talose, d-xylose, d-arabinose, l-fucose, d-glucose, d-galactose and d-fructose adopted by one enzyme, while the same series, but d-lyxose, d-arabinose and l-fucose, were accepted by the other catalyst. Disaccharides were also used: xylobiose, lactulose, sucrose, lactose and turanose with yields up to 35%. Major products due to β-1,4 or 1,6 linkage formation were observed, although minor compounds characterized by different interglycosidic linkages were also formed [64].

Convenient syntheses of interesting series of pure (β-1,4)-xylooligosaccharides were reported for xylosidase/xylanase activity present in *Thermotoga neapolitana* using various acceptors, such as 1-hexanol (producing candidates for surfactants), 9-fluorene methanol (obtaining anti-HSV agents), 1,4-butanediol (for the synthesis of new glycolipids) and geraniol (producing aroma compounds) [65].

## 7. Galactosidase

The interest of synthetic chemists for the production of α-galactosides has been present since 1970s, and it has been traced in the literature as being generally prompted by the discovery of the biological functions of these molecules. From an in-depth analysis recently published [66], a direct influence of substrate availability in enzyme discovery has been recognized. In fact, α-1,6-galactosides are more common and easily used in the investigation of substrate specificity with respect to rarer α-1,3- and α-1,4-regioisomers that have been obtained by somewhat troublesome synthetic strategies. Then, α-1,3 and α-1,4 specificity is still ascribed to few enzymes, including marine α-galactosidases.

Human gastrointestinal microflora *Bifidobacteria* possess high α-d-galactosidase activity. Galacto-oligosaccharides were synthesized with melibiose, stachyose and raffinose as starting donors. Under optimum pH for activity (pH 6.0) and high melibiose concentration (40% *w*/*v*), the biocatalyst from *Bifidobacterium bifidum* was able to form oligosaccharides with a degree of polymerization (DP) >3 and a total yield of 20.5% (*w*/*w*) [67]. The enzyme from *Bifidobacterium breve* 203 (Aga2), using melibiose as a substrate, synthesized a trisaccharide (Gal-α-1,4-Gal-α-1,6-Glc) with the novel 1,4 galactosidic link formed. In a reaction using 100 mM melibiose, approximately 11% of the trisaccharide was formed, which was isolated by a Biogel P2 column and characterized by 2D NMR spectroscopy [68].

The broad synthetic potential of the *Talaromyces flavus* α-d-galactosidase is of particular interest. The dependence of this fungal activity on the presence of organic cosolvents for the preparation of 4-nitrophenyl α-d-galactopyranosyl-(1,3)-6-*O*-acetyl-α-d-galactopyranoside has been reported [69]. In addition, the same catalyst was also used to prepare important biomolecules, such as isoglobotriose [70].

Using the common coffee beans’ α-galactosidase, methyl α-d-galactopyranosyl-(1,3)-α-d-galactopyranoside was obtained in a 51% yield in ice, while only 29% is synthesized at 37 °C [71]. The authors speculated that this is a general property of hydrolases in that they had already found the same behavior with β-galactosidases.

In one of the recent reports on the use of α-galactosidase for the synthesis of galactosides, the enzyme isolated from the crude liquid culture of *Aspergillus* sp. MK14 was able to use guar gum as a donor substrate. The guar gum is a galactomannan polysaccharide constituted by a mannan chain (β-1,4-linked) with α-galactose linked in 6-*O*-positions. Conducting the hydrolysis reaction in the presence of glycerol, galactosyl glycerol was formed at 76.6 mg/g of guar gum. Unfortunately, the authors did not investigate the regioselectivity of the transfer for this efficient reaction [72], but the possibility to use crude enzyme along with a donor polymer, which is efficiently consumed, is a very interesting asset of this enzymatic process.

A detailed and critical analysis of the literature for the production of α-galactosides with more tabular-based cross-comparisons has been already published [66] and excluded from this review, as well as the transfer of the anomer sugar β-galactose, operated by β-galactosidases, which is a much more common reaction with respect to α-galactosyl transfer. However, a study collecting different enzymes for the production of glycosylated oligosaccharides that recently appeared [73] is important. The thermophilic α-galactosidase AgaB from *Geobacillus stearothermophilus* KVE39, the β-galactosidase BglT from *Thermus thermophilus* TH125 and the mesophilic α-galactosidase RafA from *Escherichia coli* were used to realize α- and β-galactose transfers to model structures, such as sucrose, isomaltitol, and isomaltulose, studying the stereo- and regio-specificities. The products are of interest in the food industry.

## 8. Glucuronidase

Glucuronic and galacturonic acids are found in pectins and hemicelluloses (Figure 2) or in glycosaminoglycans (GAGs), in bacterial polysaccharides or in glycoconjugates resulting from detoxification reactions. Interesting enzymes are also studied for the deconjugation of glucuronides, as in the case of quercetin [74]; and biocatalyzed techniques based on UDP-sugar transferases were also studied [75] in this context.

**Figure 8:**
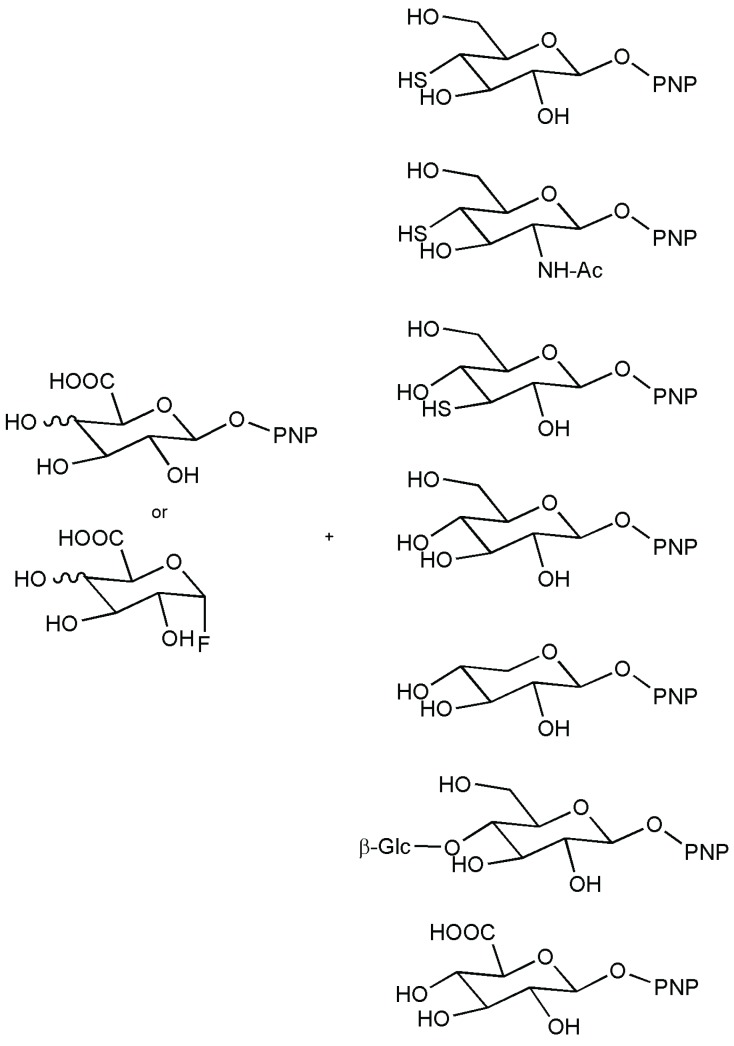
Donors and acceptors in thioglycoligase- and glycosynthase-based reactions for the synthesis of different products using *Thermotoga maritima* a β-glucuronidase [76,77].

As for the enzymes of interest in this review, in *Thermotoga maritima*, a β-glucuronidase was recently identified, cloned and expressed in *Escherichia coli*. Catalytic nucleophile identity was established by trapping the glycosyl-enzyme intermediate with 2-deoxy-2-fluoro-β-d-glucosyluronic acid fluoride, a mechanism-based inactivator. The Ala mutant Glu476Ala was then shown to be hydrolytically inactive. The acid/base catalyst was confirmed to be Glu383 by the generation and kinetic analysis of mutants modified at that position, Glu383Ala and Glu383Gln. From this knowledge, the enzyme resulted in being suitable for enzymatic synthesis by the transglycosylation mode of both wild-type or modified protein with glycosynthase and thioglycoligase approaches (see Figure 8) [76]. The enzyme is active on both glucuronides and galacturonides, and it was discovered that only using alanine mutant E383A, the transfer reaction was possible. Donors and acceptors used in these reactions are depicted in Figure 8 [77].

From *Thermotoga maritima*, an α-glucuronidase was also isolated, probably acting with the cofactor-dependent mechanism [78]. Transfer reactions seem to be unreported in this case, both in the presence of thiol- or hydroxyl-containing acceptors.

## 9. Hyaluronidase

Hyaluronic acid is a linear biopolymer consisting of glucuronic acid and *N*-acetylglucosamine linked via a β-1,4 interglycosidic linkage. Abundant in extracellular matrices, the molecule is degraded by hyaluronidases, while it is synthesized by the enzyme hyaluronan synthase. Both the excellent biocompatibility and biodegradability of fragments of hyaluronic acid are of great interest in pharmaceutics in a number of molecular devices for drug delivery and other medical and cosmetic applications [79]. A synthetic approach based on glycosaminoglycans reconstruction, using transglycosylation by hyaluronidase, has been reported also for the construction of a library of hybrid oligosaccharides [79].

All mammalian hyaluronidases can catalyze hydrolysis, as well as transglycosylation reactions of hyaluronic acid fragments. As a prototype, bovine testicular enzyme (BTH) is the commercially-available preparation that has long been considered in previous studies. In the case of BTH, the hydrolysis is favored at acidic pH values, while transglycosylations occur preferentially at neutral pH and at low NaCl concentrations. A detailed analysis of products has been reported [80] for the availability of recombinant expression systems producing purified human hyaluronidases PH-20 and Hyal-1. Interestingly, HA octasaccharide can be used as the substrate of Hyal-1 at pH 3.5, while HA hexasaccharide is generally accepted as being the shortest substrate for BTH. Quick substrate conversion was obtained between 25 μM to 1 mM; above this range, weak substrate inhibition was observed. The study of transfer reactions, selectivities and yields are all features of interest for a possible use of these enzymes in biocatalytic steps for manipulation of these biomolecules [81]. Interesting new examples of this enzyme can be derived from other natural environments. The venoms of two classes of fish, freshwater stingray (members of the genus *Potamotrygon*) and stonefish (members of the genus *Synanceia*), contain, along with proteinaceous toxins, also hyaluronidases, considered as spreading factors facilitating toxin diffusion in tissues by degrading hyaluronan. Owing to the quick enzymatic action featuring these catalysts, they can be of particular interest for biocatalysis [82].

## 10. Inulinase

**Figure 9:**
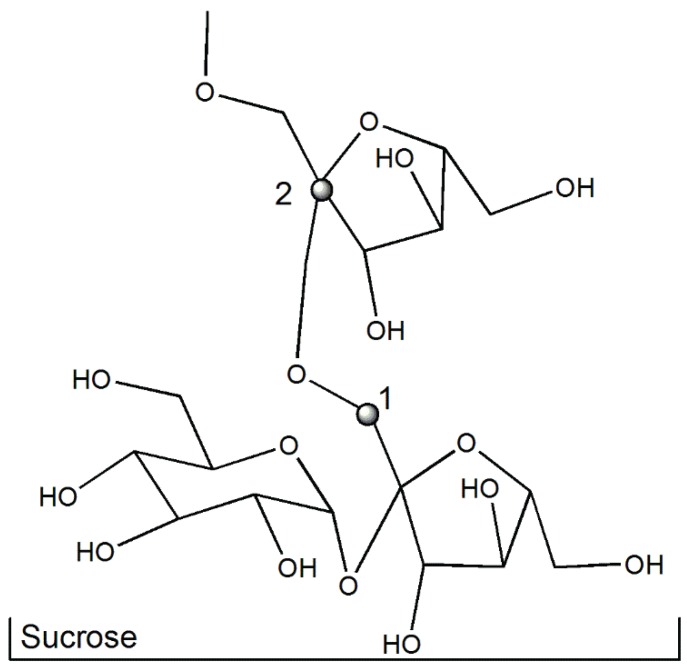
Base structure of fructo-oligosaccharides with β-(2,1)-bound fructose units.

A sucrose molecule with one or more additional fructose moieties linked to the first is the base structure of fructooligosaccharides (Figure 9). These molecules, characterized by the β-configuration of the anomeric C2 of fructose as in inulin and oligofructoses, are resistant to hydrolysis by human enzymes (glucosidase, maltase-isomaltase, sucrase) specific for α-glycosidic linkages. For this reason, oligofructoses are non-digestible oligosaccharides and can act as prebiotic agents being capable of changing colonic microbiota, boosting selective proliferation of *Bifidobacteria*. Fructooligosaccharides can be hydrolyzed by inulinases acting by endo-hydrolysis of β-d-fructosidic linkages. Inulinases are also capable of transglycosylation using inulin as a donor and sucrose as an acceptor, forming short fructooligosaccharides, such as kestose (GF2), nystose (GF3) and GF4 [83].

Other examples are reported, such as fructans [84] or levan with β-(2,6)-bound fructose units, obtained by bi-enzymatic systems [85,86].

Glycosylated sterols have been recognized as more potent agents for cholesterol lowering with respect to esterified sterols. Analytical procedures used to assess sterol profiles, including acid hydrolysis, fail to reflect the correct presence due to the drawback of acid for the potential isomerization of some components (e.g., protonation of their alkene side chain at low pH). Various glycosyl hydrolases have been evaluated for their ability to hydrolyze steryl glycosides under milder conditions. The highest activity, as demonstrated by the decrease in steryl glycosides and the increase in free sterols, was achieved using inulinase preparations. It is known that the active site containing the active glutamate residue in endoinulinase is larger than that of exoinulinase, and this is hypothesized as the enabling effect for the hydrolysis of larger substrates [87].

## 11. Sialidase

Terminal sialic residues α-linked to glycoproteins, glycolipids and polysaccharides can be hydrolyzed by sialidases (E.C. 3.2.1.18), glycosyl hydrolases that have been employed also for the synthesis of various sialyl glycoconjugates. The importance of molecules containing such structures is derived from their long-known involvement in a variety of biological processes [88]. Chemical and enzymatic synthesis of sialyloligosaccharides have been extensively reviewed by Boons and Demchenko [89]. In addition to all of the examples previously reviewed [12], a further modern one is discussed here.

**Figure 10:**
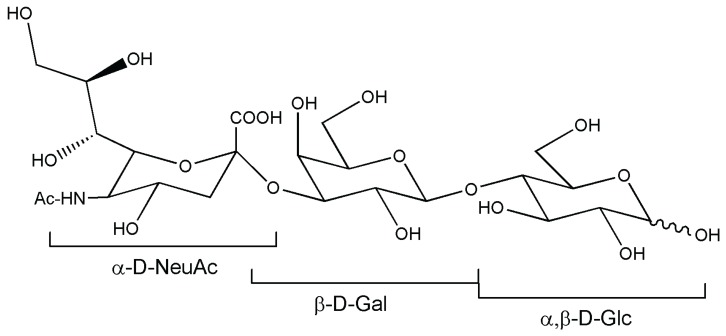
3'-sialyllactose.

3'-sialyllactose (Figure 10) represents a model case compound for human milk oligosaccharides. Enzymatic synthesis of this compound includes two components from standard procedures used in the dairy industry and a mutated sialidase derived from *Trypanosoma rangeli*, expressed in *Pichia pastoris* [90]. The process is remarkably scalable to 5 L, providing a yield of 3.6 g of 3'-sialyllactose and has been optimized by means of different immobilization techniques. Sialic acid donor substrate, casein glycomacropeptide (cGMP), was used in the form of a commercially-available product Lacprodan® (Sønderhøj, Denmark) containing 0.2 mmol/g dry matter of covalently-linked sialic acid. Interestingly, lacto-*N*-tetraose and lacto-*N*-fucopentaoses also acted as acceptor molecules [91].

## 12. Conclusions

The gross general result of the survey reported here indicates that an increasing number of new glycosyl hydrolases are appearing in the literature. Owing to the need for the manipulation of new polysaccharides, this aspect will be much more evident in the near future. Very often, natural hydrolytic activity present in wild-types at a good evolutionary selected degree of efficiency is the first to be exploited. In the current state of the art, although protein engineering is theoretically able to ensure a quick modification of proteins in different positions into synthetic, efficient catalysts, the details of each case must be known and defined with precision for a successful creation of efficient biocatalysts in synthetic mode. For this aspect, the biomolecular details of the bioprocess are needed, enabling the elaboration of data on the enzymatic characteristics (bio-), as well as on the details of the stereochemical outputs (-molecular), along with the analysis of the bioprocess (reaction conditions, solvents, yields, economic efficiency).
